# Olive oil consumption and risk of cardiovascular disease and all-cause mortality: A meta-analysis of prospective cohort studies

**DOI:** 10.3389/fnut.2022.1041203

**Published:** 2022-10-18

**Authors:** Meng Xia, Yi Zhong, Yongquan Peng, Cheng Qian

**Affiliations:** Department of Cardiology, The Affiliated Hospital of Southwest Medical University, Luzhou, China

**Keywords:** olive oil, cardiovascular disease, all-cause mortality, meta-analysis, prevention

## Abstract

**Background:**

Epidemiological studies have shown the preventive effects of olive oil consumption against cardiovascular events and all-cause deaths, but the results remain inconsistent. Herein, we performed a meta-analysis to elucidate this association.

**Materials and methods:**

A systematical literature search was conducted in online databases (PubMed and Scopus) through July 31, 2022. Prospective cohort studies providing the risk of total cardiovascular disease (CVD) or all-cause mortality for olive oil consumption were included. Relative risks (RRs) and 95% confidence intervals (CIs) were aggregated using random-effect model.

**Results:**

This meta-analysis included 13 studies comprising a total of 13 prospective cohorts. Compared with lower consumption, higher consumption of olive oil conferred a significantly reduced risk in CVD (RR: 0.85, 95% CI: 0.77–0.93, *p* < 0.001) and all-cause mortality (RR: 0.83, 95% CI: 0.77–0.90, *p* < 0.001). This beneficial effect was not modified by the potential confounders such as study country, sample size, follow-up duration, gender, and type of olive oil consumed. In dose-response meta-analysis, the summary RR of per 5-g/days increase in olive oil intake was 0.96 (95% CI: 0.93–0.99, *p* = 0.005) for CVD and 0.96 (95% CI: 0.95–0.96, *p* < 0.001) for all-cause mortality. Non-linear associations of olive oil intake with CVD and all-cause mortality were also identified (both *p* for non-linearity < 0.001), with little additional or no risk reduction observed beyond the consumption of approximately 20 g/days.

**Conclusion:**

Olive oil consumption is inversely related to the risk of CVD and all-cause mortality. Such benefits seem to be obtained with an intake of olive oil up to 20 g/days, which deserves further exploration in future studies.

## Introduction

Olive oil, an essential ingredient of the Mediterranean diet pattern, is widely used for cooking and dressing in traditional Mediterranean regions. In addition to its high content of monounsaturated fats acids (MUFAs), olive oil is abundant in other nutrients such as phenolic compounds, vitamin E, and lipid molecules that possess anti-inflammatory and antioxidant effects (1). Owing to these potential health-promoting properties, olive oil has become more popular worldwide in recent years. Dietary intake of olive oil, in particular the virgin type, has been shown to improve several cardiovascular risk factors including obesity (2), hypertension (2, 3), diabetes (4), dyslipidemia (5), endothelial dysfunction (6), and thrombosis (7). Also in the PREvención con DIeta MEDiterránea (PREDIMED) trial, a Mediterranean diet complemented with extra-virgin olive oil could lower the risk of total cardiovascular disease (CVD) by 31% as compared to a control diet (8). All these points raise the possibility that olive oil may exert protective roles against CVD and thus prevent the related deaths. Furthermore, olive oil is likely to reduce mortality through its salutary impacts on developing other chronic diseases such as certain cancers (9) and neurodegenerative disease (10).

Epidemiological studies have explored the association of olive oil intake with risk of CVD and total mortality, in either Mediterranean or non-Mediterranean populations. However, the main results from these reports were inconsistent; that is, some of them found inverse associations for total CVD (11–13) and all-cause mortality (11, 14–19), while others not (20–23). To achieve a consensus, there is a need for a comprehensive analysis of the relevant data. We, therefore, carried out a meta-analysis of prospective cohort studies to investigate the relationship between olive oil consumption and risk of CVD and all-cause mortality.

## Materials and methods

### Literature search

This study was reported based on the Meta-analysis of Observational Studies in Epidemiology guideline (24). We performed an electronic literature search in databases of PubMed and Scopus from inception to July 31, 2022, using the search terms as follows: “olive oil,” “CVD,” “mortality,” and “cohort study.” The detailed search algorithm is exhibited in Supplementary Table 1. To identify additional pertinent studies, the bibliographies of retrieved articles and reviews were also scrutinized. There was no restriction to the language of publications.

### Inclusion criteria

Studies were considered as eligible if all of the following were achieved: (1) prospective cohort design; (2) the exposure of interest was olive oil consumption; (3) the recorded outcomes were total CVD or all-cause mortality; (4) have provided the risk measures such as relative risk (RR), hazard ratio, or odds ratio. To be included in dose-response meta-analysis, studies should present the risk estimates for ≥3 quantitative ranges of olive oil consumption. Reviews, comments, editorials, and studies only including specific CVD outcomes (e.g., coronary heart disease or stroke) were excluded. For publications pertained to the same cohort, we retained the version with the longest follow-up time.

### Data collection and quality assessment

The general features of eligible studies were extracted, including study author, year of publication, cohort name and country, sample size, age range, gender, ascertainment methods for exposure and outcome, mean follow-up duration, and risk estimates and confounders in the maximally adjusted model. The Newcastle-Ottawa Scale (NOS) (25) was utilized to determine the quality of studies. This scale scored each included study a maximum of nine points, and high-quality studies were defined as those with NOS scores ≥7. The above methodological steps were accomplished by two independent reviewers (MX and YZ), and discordances were resolved by consulting with a third reviewer (YP).

### Statistical analysis

The summary risk measure of outcome in this study was reported as RR and its 95% confidence interval (CI). For studies that only provided hazard ratio or odds ratio, we considered it as equal to RR. The study-level RRs for the highest vs. lowest consumption of olive oil were combined using random-effect models. Heterogeneity across the included studies was examined with the Cochrane *Q*-test and the *I*^2^ statistic, with *I*^2^ > 50% reflecting the presence of significant heterogeneity. Subgroups analyses were conducted according to study information including study country (Mediterranean or non-Mediterranean), number of participants (<10,000 or ≥10,000), follow-up duration (<10 years or ≥10 years), gender (male or female), and olive oil type (virgin or common). Between-subset differences were checked by implementing a heterogeneity test across subsets instead of across studies (26). To measure the influence of single study, we also carried out a sensitivity analysis by removal of each report one at a time. When the number of included studies is ≥10, publication bias of the pooled estimate was detected using AS-Thompson test (27).

In addition, dose-response meta-analyses were performed to further investigate the association of olive oil intake with the risk estimates of events. For each study, the median or mean olive oil intake for every category was allocated to each corresponding RR. If the median or mean values were not provided, we estimated them as described in previous studies (28). A linear trend of the RRs across categories of olive oil consumption for individual studies was firstly calculated following the method proposed by Greenland and Longnecker (29) and Orsini et al. (30). The study-specific RRs with 95% CIs for a 5-g/days increase in olive oil intake were aggregated using random-effect meta-analysis. To examine the potential non-linear dose-response pattern, we next developed a restricted cubic spline model using three knots at the 10th, 50th, and 90th percentiles of olive oil intake. A *p*-value for non-linearity was computed by testing the null hypothesis that the coefficient of the second spline is equal to zero.

All statistical strategies were realized with Review Manager 5.3 (The Cochrane Collaboration, Oxford, UK) and R 4.1.0 (The R Foundation for Statistical Computing, Vienna, Austria) software. Results were considered as of significance if the corresponding *p*-value was <0.05.

## Results

### Included studies and general features

**FIGURE 1:**
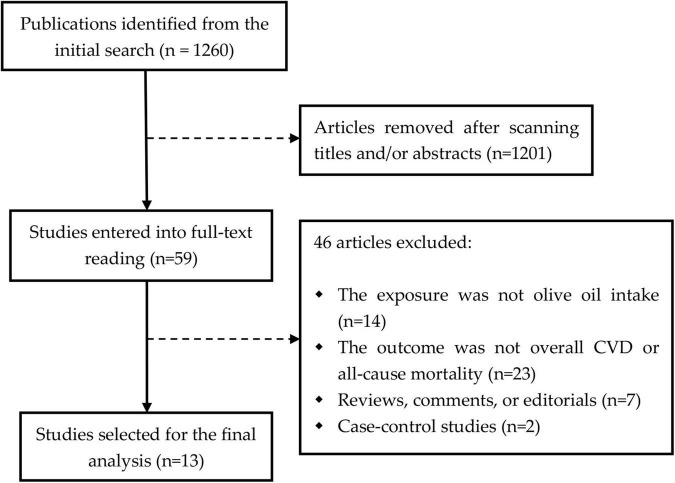
The flowchart of study searching process. CVD, cardiovascular disease.

We initially obtained 1,260 publications using the predefined search strategies. After scanning the titles/abstracts, 59 articles were selected for full-text reviewing, of which 46 papers were further excluded because of failure to meet the eligible criteria. As a result, a total of 13 prospective cohorts from 13 reports (11–23) were included (Figure 1). These studies were published between 2003 and 2022, with follow-up intervals ranging from 4 to 28 years. Most of studies collected the dietary data on olive oil intake based on food-frequency questionnaires, and the outcome events were identified using International Classification of Diseases codes or other medical records. The main characteristics of the eligible studies were summarized in Table 1. Besides, all of the studies were assigned a NOS score of ≥7, indicating the evidence of high methodological quality.

**TABLE 1:** General characteristics of the included cohort studies.

Author, year	Cohort, country	Sample size	Age (years)	Men (%)	Ascertainments		FU (years)	Adjustments	NOS score
					Olive oil	Outcomes			
Atkins et al. (11)	BRHS, UK	3,328	60–79	100	FFQ	ICD-9 codes	11.3	Age, energy intake, smoking, alcohol, PA, social class, BMI, and a modified version of healthy dietary score	8
Barzi et al. (14)	GISSI, Italy	11,246	19–90	85	FFQ	ICD-9 codes	6.5	Age, sex, hypertension, HDL-c, diabetes, smoking, claudication, electrical instability, left ventricular dysfunction, residual myocardial ischemia, dietary supplementation, pharmacological therapies, and foods	7
Buckland et al. (15)	EPIC, Spain	40,622	29–69	38	Dietary history records	ICD-9 and ICD-10 codes	13.4	Age, sex, study center, energy intake, BMI, waist circumference, education, smoking, alcohol, PA, and intake of fruit, vegetables, meat, and dairy	8
Chrysohoou et al. (16)	Ikaria, Greece	673	65–100	49	FFQ	ICD codes	4	Age, sex, smoking, PA, CVD history, pulse pressure, education, depression level, comorbidities, and Mediterranean diet score	7
Donat-Vargas et al. (20)	EPIC, Spain	39,393	29–69	37	Dietary history records	ICD-9 and ICD-10 codes	22.8	Age, sex, study center, energy intake, education, smoking, alcohol, HC, hypertension, diabetes, PA, BMI, and intake of dietary fiber, fruits, vegetables and sodium	8
	SUN, Spain	18,266	18–91	40	FFQ	Questionnaires and medical records	10.8	Age, sex, year of recruitment, energy intake, education, smoking, alcohol, PA, BMI, HC, blood pressure, hypertension, diabetes, and intake of dietary fiber, fruits, vegetables and sodium	9
Guasch-Ferré et al. (12)	HPFS, NHS, USA	92,978	25–75	34	FFQ	Self-reports and medical records	24	Age, race, ancestry, energy intake, smoking, alcohol, PA, family history of diabetes, myocardial infarction and cancer, HC, hypertension, diabetes, use of multivitamin, aspirin, postmenopausal status and menopausal hormone use (women), and intake of red meat, fruits, vegetables, nuts, soda, whole grains and *trans* fat	7
Guasch-Ferré et al. (17)	HPFS, NHS, USA	92,383	30–75	34	FFQ	Self-reports and medical records	28	Age, race, ancestry, energy intake, marital and living status, smoking, alcohol, BMI, PA, family history of diabetes, myocardial infarction and cancer, HC, hypertension, diabetes, use of multivitamin, aspirin, postmenopausal status and menopausal hormone use (women), and intake of red meat, fruits, vegetables, nuts, soda, whole grains and *trans* fat	7
Guasch-Ferré et al. (13)	PREDIMED, Spain	7,216	55–80	43	FFQ	Questionnaires and medical records	4.8	Age, sex, energy intake, intervention group, BMI, smoking, alcohol, education, PA, diabetes, hypertension, HC, antihypertensive medication and statins, and Mediterranean diet score	8
Letois et al. (18)	Three-City, France	8,937	≥65	39	FFQ	ICD-10 codes	9	Sex, study center, education, income, occupation, smoking, alcohol, history of CVD, BMI, depression, diabetes, hypertension, HC, dependence, self-rated health and diet quality, number of drugs, and number of chronic diseases	8
Kouli et al. (21)	ATTICA, Greece	2,020	18–89	50	FFQ	ICD codes	8.4	Age, sex, BMI, smoking, PA, education, HC hypertension, diabetes, and metabolic syndrome	7
Sadeghi et al. (22)	ICS, Iran	5,432	≥35	49	FFQ	Medical records	11.3	Age, sex, education, residency, smoking, BMI, PA, family history of CVD, diabetes, hypertension, HC, aspirin use, postmenopause in women, energy intake, and intake of red meat, fish, fruit, vegetable, other cooking oil, animal fats, fast food, cereals, legumes, nuts and seeds, sweets, soft drink and beverages	9
Trichopoulou et al. (23)	EPIC, Greek	22,043	20–86	40	FFQ	ICD-9 codes	3.7	Age, sex, waist-to-hip ratio, energy-expenditure score, education, smoking, BMI, and energy intake	9
Zhang et al. (19)	NIH-AARP, USA	521,120	50–71	57	FFQ	ICD-9 and ICD-10 codes	16	Age, sex, race, energy intake, BMI, education, marital status, household income, smoking, PA, usual activity at work, perceived health condition, history of heart disease, stroke, diabetes and cancer, Healthy Eating Index-2015, and consumption of other cooking oils	9

BRHS, British Regional Heart Study; BMI, body mass index; CVD, cardiovascular disease; EPIC, European Prospective Investigation into Cancer and Nutrition; FFQ, food-frequency questionnaire; FU, follow-up; GISSI, Gruppo Italiano per lo Studio della Sopravvivenza nell’Infarto Miocardico; HC, hypercholesterolemia; HDL-c, high-density lipoprotein cholesterol; HPFS, Health Professionals Follow-up Study; ICD, International Classification of Diseases; ICS, Isfahan Cohort Study; MI, myocardial infarction; NA, not applicable; NIH-AARP. National Institutes of Health-American Association of Retired Persons; NHS, Nurses’ Health Study; NOS, Newcastle–Ottawa Scale; PA, physical activity; PREDIMED, PREvención con DIeta MEDiterránea; SUN, Seguimiento Universidad de Navarra.

### Risk of cardiovascular disease

**FIGURE 2:**
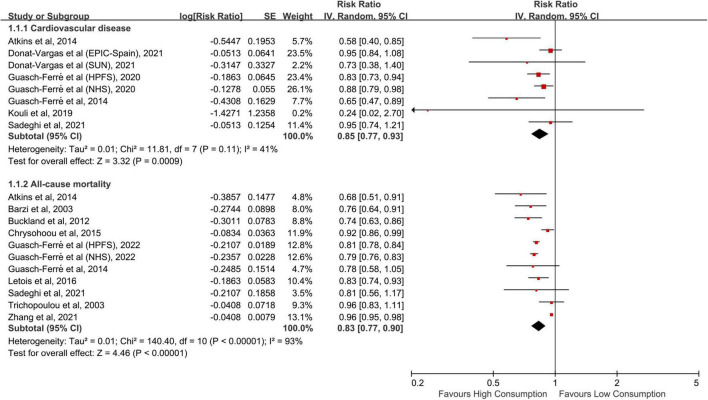
Meta-analytic results for the highest vs. lowest comparison of olive oil consumption. CI, confidence interval; SE, standard error.

Eight cohorts from six studies (11–13, 20–22) were included in the meta-analysis of CVD risk, totaling 261,016 patricians and 14,033 CVD cases. The pooled RR for the highest vs. lowest consumption of olive oil was 0.85 (95% CI: 0.77–0.93, *p* < 0.001; Figure 2), without substantial heterogeneity across the studies (*I*^2^ = 41%, *p* = 0.107). In subgroup analyses, we found no significant differences between strata of study region, sample size, follow-up duration, sex, and olive oil type (Table 2). The combined risk estimate of CVD was not altered in the sensitivity analysis by omitting each study one at a time.

**TABLE 2:** Subgroup analyses for the risk of cardiovascular disease and all-cause mortality.

Subgroup	Cardiovascular disease					All-cause mortality				
	No. of cohorts	*I* ^2^	RR (95% CI)	*p*	*p* for interaction	No. of cohorts	*I* ^2^	RR (95% CI)	*p*	*p* for interaction
**Country**										
Mediterranean	5	38%	0.86 (0.72–1.03)	0.09	0.70	7	50%	0.84 (0.78–0.92)	<0.001	0.80
Non-Mediterranean	3	54%	0.82 (0.72–0.94)	0.005		4	98%	0.83 (0.73–0.94)	<0.001	
**No. of participants**										
<10,000	4	56%	0.72 (0.53–0.97)	0.03	0.19	5	37%	0.85 (0.77–0.93)	<0.001	0.87
≥10,000	4	0%	0.88 (0.82–0.95)	<0.001		6	96%	0.84 (0.75–0.93)	0.001	
**Follow-up duration**										
<10 years	2	0%	0.64 (0.47–0.88)	0.006	0.07	5	44%	0.87 (0.80–0.94)	<0.001	0.33
≥10 years	8	33%	0.87 (0.80–0.95)	0.002		6	96%	0.81 (0.72–0.91)	<0.001	
**Gender**										
Male	2	67%	0.73 (0.52–1.02)	0.07	0.29	5	94%	0.89 (0.80–0.99)	<0.001	0.57
Female	1	NA	0.88 (0.79–0.98)	0.02		4	95%	0.85 (0.75–0.96)	<0.001	
**Type of olive oil**										
Virgin	2	68%	0.74 (0.53–1.04)	0.08	0.19	2	0%	0.92 (0.86–0.99)	0.04	0.56
Common	2	32%	0.95 (0.81–1.12)	0.56		2	45%	0.98 (0.82–1.15)	0.77	

CI, confidence interval; NA, not applicable; RR, relative risk.

### Risk of all-cause mortality

A total of 11 independent cohorts from 10 publications (11, 13–19, 22, 23) have reported the risk of all-cause mortality, comprising 713,000 subjects and 173,817 deaths. Compared with the lowest intake, the highest intake of olive oil was related to an obvious decrease in the risk of all-cause mortality (RR: 0.83, 95% CI: 0.77–0.90, *p* < 0.001; Figure 2), with substantial between-study heterogeneity (*I*^2^ = 93%, *p* < 0.001). Excluding each report in sequence had no influence on the pooled result. The combined RRs were similar between subsets stratified by the aforementioned features (Table 2). Moreover, there was no indication of publication bias from the AS-Thompson test (*p* = 0.175).

### Dose-response relationship

**FIGURE 3:**
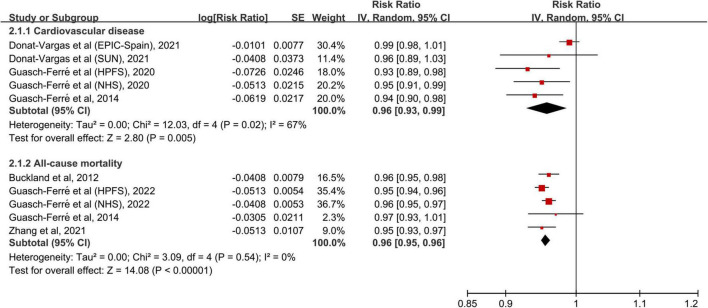
Meta-analytic results for each 5-g/days increase in olive oil consumption. CI, confidence interval; SE, standard error.

**FIGURE 4:**
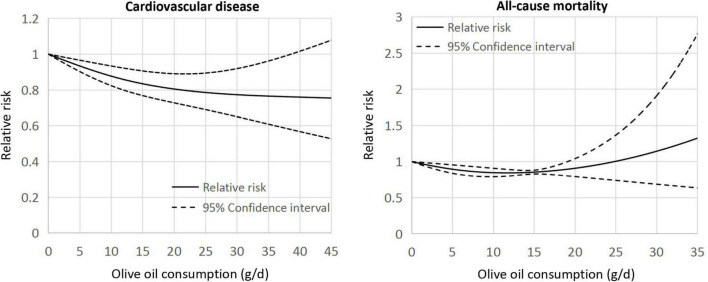
Curvilinear associations of olive oil consumption with the risk of cardiovascular disease and all-cause mortality.

The dose-response meta-analysis included five cohorts (12, 13, 20) for CVD and five cohorts (13, 15, 17, 19) for all-cause mortality. In linear dose-response pattern, the aggregated RR of CVD for each 5-g/days increment in olive oil consumption was 0.96 (95% CI: 0.93–0.99, *p* = 0.005; Figure 3). Similar result was found in the pooling analysis of all-cause mortality (RR: 0.96, 95% CI: 0.95–0.96, *p* < 0.001). In addition, a curvilinear association was seen of olive oil intake with CVD and all-cause mortality (both *p* for non-linearity <0.001; Figure 4). For CVD events, the trend of risk reduction appeared to be largely attenuated when consuming olive oil ≥20 g/days. For all-cause death, there seemed no preventive effects when increasing the intake level beyond 20 g/days.

## Discussion

In this meta-analysis, we observed reduced risks for CVD and all-cause mortality in the comparison of the highest vs. lowest consumption of olive oil. These findings were consistent in the subanalyses by study region, sample size, follow-up duration, gender, and type of olive oil. Linear dose-response analysis indicated that both the risk of CVD and all-cause mortality was lowered by 4% for every 5-g/days increase in olive oil intake. However, a curvilinear dose-response pattern was documented; when consuming olive oil exceeding 20 g/days, there appeared little further decrease for the risk of CVD, with no preventive benefits for all-cause mortality.

In line with our findings, two published meta-analyses of observational studies (31, 32) have also reported the inverse association of olive oil consumption with stroke incidence and all-cause deaths. Both of them were restricted in a limited number of prospective cohorts, and neither, however, undertook a clear evaluation on a composite of total cardiovascular events. Furthermore, categories of olive oil use differed across the included cohorts, potentially complicating the interpretation of the pooled results. For this aspect, a dose-dependent meta-analysis may represent an alternative to handle this problem (33), but none of the previous studies (31, 32) have explored the dose-response pattern. The present meta-analysis incorporated the newly published data from several large longitudinal cohorts (12, 17, 19, 20), thus providing more comprehensive and reliable evidence for the cardiovascular and survival benefits in relation to olive oil consumption.

Olive oil delivers high amounts of favorable nutrients including MUFAs in the form of oleic acid, polyphenols, vitamin E, and lipid molecules. These nutritional metabolites endow olive oil, especially the extra-virgin one, with cardiometabolic protective effects via the well-known anti-inflammatory, antioxidant, and anti-thrombotic functions (34). Accordingly, olive oil has been proven to improve a range of cardiometabolic parameters such as lipid profile, blood pressure, insulin sensitivity, and glycemic control in recent dietary feeding trials (35, 36). Olive oil shows great potentials in positively modulating the gut microbiota composition and microbially produced products, which has been linked to the prevention of cardiometabolic diseases (37). As for the individual components of olive oil, dietary intake of MUFAs and polyphenols were correlated with a lower risk of CVD (38) and/or all-cause mortality (39, 40). Together, the above mechanisms may contribute to the observed inverse association of olive oil with CVD events and total mortality.

Some issues need to be emphasized in our work. Despite inclusion of high-quality studies, substantial heterogeneity was detected in the categorical meta-analysis for all-cause mortality. This may be attributed to the variances in the intake levels of olive oil across studies, since we recorded no heterogeneity in the linear dose-dependent analysis. Due to the high-calorie content, persons eating more olive oil tend to have less intake of other macronutrients to keep a energy balance. This point may be pertinent to the risk of CVD and mortality which are often observed to be increased among individuals with higher intakes of carbohydrate (41). Indeed, only three included studies (14, 16, 21) have not adjusted for total energy intake and exclusion of them showed no influence on our pooled results (data not shown), which largely reduced this possible source of confounding. In addition, the healthy benefits appeared to be more pronounced in virgin than in common olive oil consumers, although no statistically significance was achieved in our subgroup analyses. Of the eligible cohorts most did not provide the specific information about the consumed type of olive oil, making it difficult to detect the potential differences. Common olive oil is a mixture of virgin and refined oil, and the latter usually accounts for ≥80% of the total volume. In contrast to the virgin one, the common variety of olive oil has fewer bioactive compounds (e.g., polyphenols) because most of them are potentially damaged and chemical solvents are added during the refining process (42). Finally, we found a non-linear relationship between olive oil intake and risk of CVD and all-cause mortality, where the benefits of olive oil were weakened with a consumption dose of ≥20 g/days. Such a dose-response pattern was also seen in a previous meta-analysis (4) showing a risk reduction in type 2 diabetes with intake of olive oil up to 15–20 g/days. It seems biologically plausible that a saturation effect may be present in the dose-dependent association, but to date we really do not know the specific underlying mechanisms. However, the observed curvilinear correlation should also be interpreted with caution, since we included a limited number of cohorts in dose-response analysis and the potential residual confounding cannot be completely ruled out. Further studies are warranted to illuminate if there is a threshold beyond which no more advantage is likely to be obtained from olive oil consumption.

The limitations of this meta-analysis should not be neglected. Firstly, the recall and selection bias resulting from the observational context of original studies cannot be fully eliminated. However, we included only the risk estimates derived from the maximally adjusted model in prospective cohort studies, which greatly attenuated this bias. Secondly, due to the insufficient data, it is impossible to make a clear conclusion on the interaction of the pooled RRs with some important confounders such as patients’ age, gender, body mass index, and the variety of olive oil. Thirdly, the majority of the cohorts were from the United States and Europe; thus, we should be prudent when extrapolating the findings to other populations.

In summary, olive oil consumption was associated with reduced risks of CVD events and all-cause mortality. Consuming olive oil up to 20 g/days may be optional, with no more benefits provided beyond this threshold. These results support the current dietary recommendations to increase the intake of olive oil instead of other fats for improving human health and longevity. Future prospective studies are required to further depict the dose-dependent cardiovascular and survival effects in relation to olive oil consumption.

## Data availability statement

The original contributions presented in this study are included in the article/Supplementary material, further inquiries can be directed to the corresponding author.

## Author contributions

CQ conceived and designed the meta-analysis. MX and YZ performed the meta-analysis and analyzed the data. YP contributed materials and analysis tools. MX wrote the manuscript, with key intellectual contents revised by CQ. All authors have read and approved the final version of manuscript.
