# Supplementary material for: Olive oil consumption and risk of cardiovascular disease and all-cause mortality: A meta-analysis of prospective cohort studies
Source: Front Nutr. 2022 Oct 18;9:1041203. doi: 10.3389/fnut.2022.1041203 (PMC9623257; doi:10.3389/fnut.2022.1041203)
Supplement: Supplementary file 1 [file Table_1.DOCX]

Supplementary Table 1. Detailed search strategies (for PubMed)

| **Search line** | **Search term** | **No. of**  **Hits** |
| --- | --- | --- |
| #1 | (olive oil[MeSH Terms]) OR (olive oil) | 13,573 |
| #2 | ((cardiovascular diseases[MeSH Terms]) OR (cardiovascular disease)) OR (cardiovascular event) | 2,862,327 |
| #3 | ((cerebrovascular disorders[MeSH Terms]) OR (cerebrovascular disease)) OR (cerebrovascular event) | 445,894 |
| #4 | (((((((mortality[MeSH Terms]) OR (death[MeSH Terms])) OR (mortality)) OR (death)) OR (survival)) OR (survive)) OR (fatal)) OR (dead) | 3,298,648 |
| #5 | #2 OR #3 OR#4 | 5,602,294 |
| #6 | (((((((cohort studies[MeSH Terms]) OR (cohort)) OR (longitudinal)) OR (follow-up)) OR (prospective)) OR (hazard ratio)) OR (relative risk)) OR (risk ratio) | 5,712,974 |
| #7 | #1 AND #5 AND #6 | 953 |
